# Postinsertional Cable Movements of Cochlear Implant Electrodes and Their Effects on Intracochlear Pressure

**DOI:** 10.1155/2016/3937196

**Published:** 2016-11-09

**Authors:** I. Todt, D. Karimi, J. Luger, A. Ernst, P. Mittmann

**Affiliations:** Department of Otolaryngology, Head and Neck Surgery, Unfallkrankenhaus Berlin, Berlin, Germany

## Abstract

*Introduction.* To achieve a functional atraumatic cochlear implantation, intracochlear pressure changes during the procedure should be minimized. Postinsertional cable movements are assumed to induce intracochlear pressure changes. The aim of this study was to observe intracochlear pressure changes due to postinsertional cable movements.* Materials and Methods.* Intracochlear pressure changes were recorded in a cochlear model with a micro-pressure sensor positioned in the apical region of the cochlea model to follow the maximum amplitude and pressure gain velocity in intracochlear pressure. A temporal bone mastoid cavity was attached to the model to simulate cable positioning. The compared conditions were (1) touching the unsealed electrode, (2) touching the sealed electrode, (3) cable storage with an unfixed cable, and (4) cable storage with a fixed cable.* Results.* We found statistically significant differences in the occurrence of maximum amplitude and pressure gain velocity in intracochlear pressure changes under the compared conditions. Comparing the cable storage conditions, a cable fixed mode offers significantly lower maximum pressure amplitude and pressure gain velocity than the nonfixed mode.* Conclusion.* Postinsertional cable movement led to a significant pressure transfer into the cochlea. Before positioning the electrode cable in the mastoid cavity, fixation of the cable is recommended.

## 1. Introduction 

Intracochlear structural and functional preservation is the aim of modern cochlear implantation. Structural effects are significantly decreased by the development of atraumatic electrodes. Functional preservation is assumed to be highly dependent on the surgical technique used due to intersurgeon variability.

Besides electrode design, the insertion angle, intracochlear size, insertional force, tip size, and application of protective agents are factors that have suggested to contribute to the preservation of residual hearing [1]. A different view in the field supports the minimization of intracochlear pressure (ICP) during the cochlear implant procedure [2]. Experimentally, it has been shown that different steps during the procedure affect the occurrence of pressure. Preinsertional factors are openings of the round window [3], the size of the round window opening [4], and transfluid opening of the round window [5]. The development of atraumatic opening of the round window has led to the development of specific opening tools [6]. Insertional factors have been observed experimentally under force aspects [7] and even under the point of pressure occurrence in terms of speed [8], moisturized insertions [4], and tremor aspects [9]. Different studies have shown the clinical relevance of these findings [10–12].

Postinsertional pressure factors occur after the positioning of the electrode intracochlearly. Two factors can be discussed, that is, sealing-related changes, which differ significantly depending on the method of sealing the electrode to the cochlea [13], and cable movements related to touching the inserted array and positioning the electrode cable in the mastoid cavity.

Attaching a floating mass transducer to the cochlear implant cable causes laser Doppler vibrometric measured output level in the range of 80–93 dB [14, 15]. Therefore, a traumatic level of intracochlear pressure changes cannot be excluded by moving the electrode cable.

The aim of the present study was to observe the effect of postinsertional cable touching and cable movements on intracochlear pressure changes in a model.

## 2. Material and Methods

### 2.1. Model and Insertion Techniques

#### 2.1.1. Pressure Sensor

The ICP was measured using a micro-optical pressure sensor FOP (FISO, Canada). Basically, the tip of the pressure sensor is a hollow glass tube sealed on one end by a thin plastic film diaphragm coated with a reflective surface of evaporated gold. The optical fiber is located in the glass tube with a small distance (50–100 *μ*m) to the diaphragm tip. The optical fiber is attached to a LED light source and to a photodiode sensor. Light from the LED source reaches the sensor tip of the optical fiber, fans out as it exits the fiber, and is reflected by the gold-covered flexible diaphragm. The reflected light is sensed by the photodiode. Small pressure induced distance displacements of the diaphragm modulate the intensity of reflected light. The sensor is connected to a module that is linked to a computer. Evolution software was used to record the ICP. The time sensitivity of the sensor was 300 measurements per second.

#### 2.1.2. Model

The model was a full-scale model of the cochlea with a volume of 87 mm^3^, which is slightly above the physiological range [16]. The sensor was positioned through a drilled hole in the apical region of the cochlea. The sensor was fixed in its position with fibrin glue. The sensor was placed within the channel in such a way that the tip was not in contact with the edge of the channel or the ground. Afterwards, the cochlea was microscopically controlled to exclude any enclosed air bubbles. The experiments were in series with a sensor in an unchanged position to exclude sensor position-related bias and to allow interexperimental comparability.

#### 2.1.3. Model Set-Up

1. The electrode is not sealed. The electrode is touched by a needle.
2. The electrode is postinsertionally sealed with fat. The electrode is touched by a needle.
3. and (4)) A human temporal bone is placed behind the artificial cochlear model to simulate the posterior tympanotomy and mastoid size. The inserted electrode is sealed with fat. For (4), fibrin glue is placed in the posterior tympanotomy to fix the electrode cable.
4. All experiments were performed five times for each condition. An Advanced Bionics HFMS electrode was used.

#### 2.1.4. Analysis

Statistically, the maximum amplitude of pressure change was calculated and statistically analyzed by one-way ANOVA and the Tukey post hoc test (SPSS 10.00). Additionally, the angular speed was estimated and statistically analyzed by one-way ANOVA and the Tukey post hoc test (SPSS 10.0).

This study was approved by the institutional review board (*IRB-ukb-HNO-2016/03*).

#### 2.1.5. Experiments

1. Touching the inserted electrode without sealing. The electrode was touched in a manner similar to the postinsertional fascia positioning procedure.
2. Touching the inserted electrode after sealing.
3. The electrode was touched in a manner similar to the postinsertional fascia positioning procedure when the fascia is placed.
4. Positioning the cable of the inserted and sealed electrode in the mastoid cavity without fixation.
5. The cable of the electrode was not fixed in the posterior tympanotomy.
6. Positioning the cable of the inserted and sealed electrode in the mastoid cavity with fixation.
7. The cable of the electrode was fixed in the posterior tympanotomy with fibrin glue.

## 3. Results

**Figure 1:**
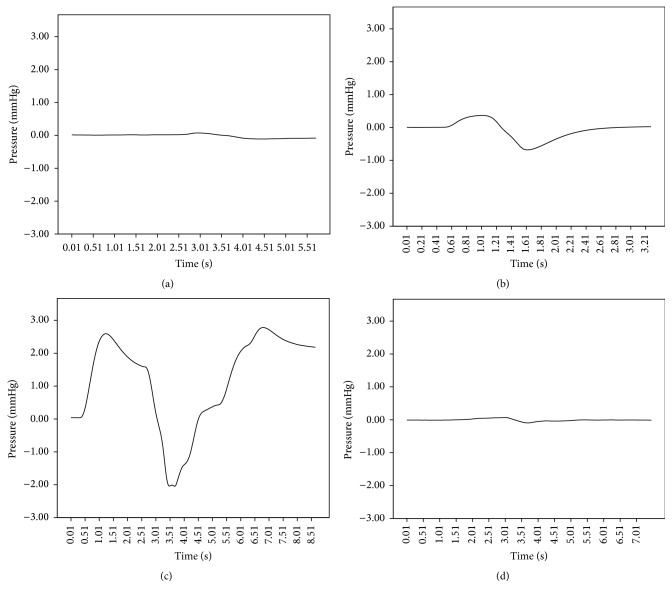
Exemplary pressure changes for the conditions (a) unsealed, (b) sealed, (c) unfixed, and (d) fixed.

Exemplary presentations of the ICP related to the different procedures are presented in Figures 1(a), 1(b), 1(c), and 1(d).

### 3.1. Measurement of Maximum Amplitude Changes (Figure 2)

A one-way ANOVA was conducted to determine if the maximum amplitude changes were different among each postinsertional movement group. Postinsertional movements were classified into four groups: touching without a patch (*n* = 5), touching with a patch (*n* = 5), positioning without fixation (*n* = 5), and positioning with fixation in the posterior tympanotomy (*n* = 5). Data are presented as the mean in mmHg ± standard deviation and Pascal (PA). Maximum amplitude changes were statistically significantly different between the conditions,* F*(3, 16) = 8.353, *p* = 0.001. Maximum amplitude changes increased from touching without a patch (0.12 ± 0.1) (15.96 ± 13.3 PA), to positioning with fixation in the posterior tympanotomy (0.23 ± 0.1) (30.59 ± 13.3 PA), to touching with a patch (1.11 ± 0.9) (147.63 ± 119.7 PA), to positioning without fixation (7.02 ± 5) (933.66 ± 665 PA), in that order.

#### 3.1.1. Statistical Analysis

The Tukey post hoc analysis revealed that the increase from positioning without fixation to positioning with fixation in the posterior tympanotomy (6.79, 95% CI (2.18 to 11.4), *p* = 0.003) was statistically significant. No other group differences were statistically significant.

### 3.2. Measurement of Pressure Gain Velocity (Figure 3)

To determine if the pressure gain velocity was different among each postinsertional movement group, a one-way ANOVA was conducted. Postinsertional movements were classified into the same four groups. Data are presented as mean in mmHg/s ± standard deviation. Pressure gain velocity was statistically significantly different between the conditions,* F*(3, 16) = 7.144, *p* = 0.003. Maximum pressure gain velocity increased from touching without a patch (0.08 ± 0.1), to positioning with fixation in the posterior tympanotomy (0.48 ± 0.4), to touching with a patch (1.14 ± 0.6), to positioning without fixation (5.9 ± 4.5), in that order.

#### 3.2.1. Statistical Analysis

The Tukey post hoc analysis revealed that the increase from positioning without fixation to positioning with fixation in the posterior tympanotomy (5.47, 95% CI (1.35 to 9.6), *p* = 0.008) was statistically significant. No other group differences were statistically significant.

## 4. Discussion

Pathophysiologically relevant acoustic levels lead to large static ICP changes or fast pressure changes with a high angular speed [17, 18]. The insertion of a cochlear implant electrode into the cochlea leads to the displacement of fluid and causes pressure changes [2]. This observation led to the question of a possible impact of the insertion procedure on the ICP, which may contribute to the loss of residual hearing. Although preinsertional and insertional factors affecting the intracochlear pressure have been shown to be responsible for the loss of residual hearing, postinsertional factors have been studied less.

Recently, two publications showed hearing sensation effects by coupling a floating mass transducer to an inserted cochlear implant electrode measured by laser Doppler vibrometry and showed anecdotally that cable movements might be responsible for the postinsertional loss of EcochG [14, 15]. We observed in our study significant increases in pressure after touching an inserted cochlear implant electrode, whether it is sealed or not (15.9 PA to 147.6 PA). The observed values transferred into dB are in the range of 118 dB to 137 dB, indicating the importance of handling the inserted electrode as carefully as possible, because it acts like a loudspeaker in the cochlea. Even higher values were observed in intracochlear pressure changes in association with cable positioning into the mastoid cavity. We observed values for an unfixed cable and for a fixed cable of 30.6 PA and 933.6 PA for the amplitude maximum and 0.5 mmHg/s and 5.9 mmHg/s for the pressure gain velocity, respectively. Transferring the measured PA values into dB, we observed a mean maximum of 153.4 dB for the unfixed cable positioning condition. Since in our experiments the cable did not “spring” or “flop,” as sometimes occurs during surgery, the PA values for a springing cable can be assumed to be higher.

Although the measured conditions were artificial, using a model, clinical relevance is highly probable since the measured values are, in comparison with other studies [3–5, 8, 9], by far the highest. Importantly, our mean maximum pressure amplitude values of 933.6 PA up to 1598 PA are above the measured pathologically relevant level in the guinea pig of +700 PA [17]. Therefore, an impact on the functionality of the cochlea due to pressure changes induced by cable movements can be assumed.

A solution for the problem of pressure transfer through the electrode is provided in experiment 4. Here, we observed a significant decrease in pressure by fixing the cable in the posterior tympanotomy.

## 5. Conclusion

Postinsertional cable movements lead to pressure transfer into the cochlea. Based on our model experiments, touching the inserted electrode should be minimized. Before positioning the electrode cable in the mastoid cavity, fixation in the posterior tympanotomy is highly recommended.

## Figures and Tables

**Figure 2:**
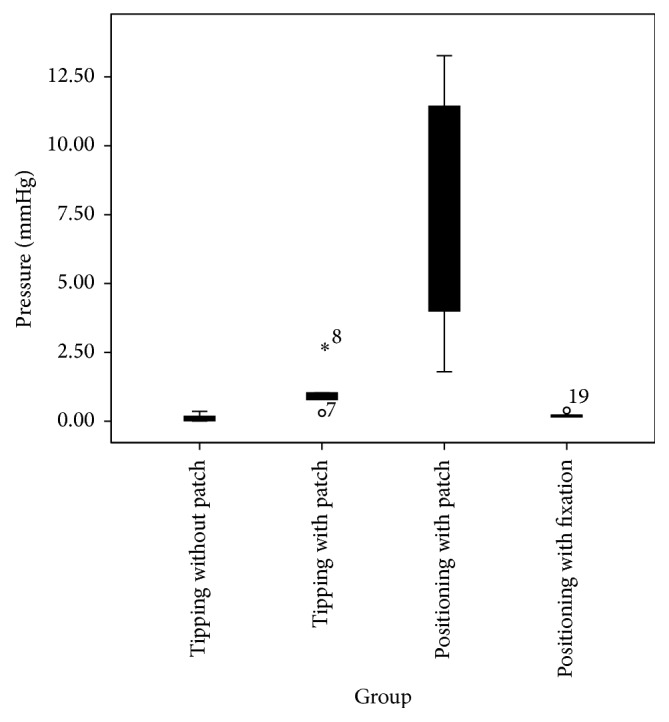
Comparison of* t* ICP maximum changes. “*∗*” and “∘” refer to outliers. Outliers are included in the statistical calculation.

**Figure 3:**
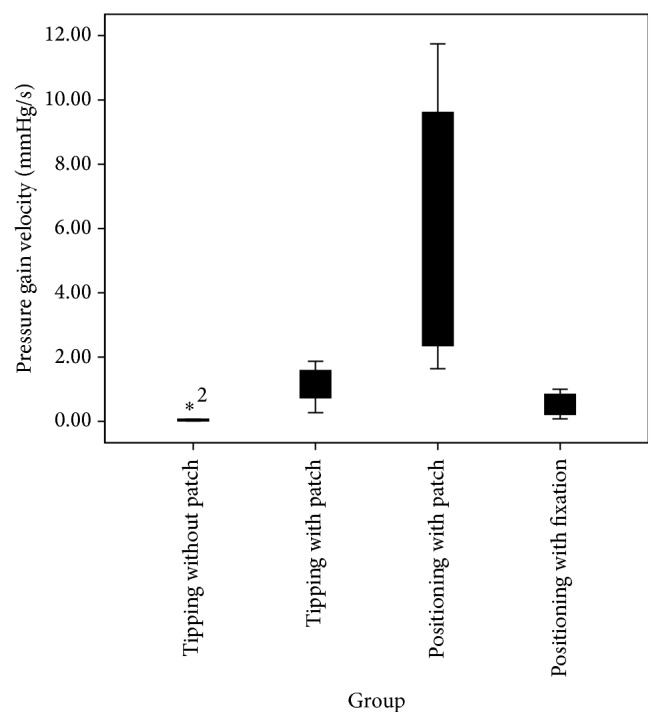
Comparison of maximum pressure gain velocity related to ICP changes. “*∗*” refers to outliers. Outliers are included in the statistical calculation.
